# Low interface state density and large capacitive memory window using RF sputtered NiO nanoparticles decorated MgZnO thin film

**DOI:** 10.1038/s41598-025-86395-z

**Published:** 2025-01-17

**Authors:** Mritunjay Kumar, Jay Chandra Dhar

**Affiliations:** Department of Electronics and Communication Engineering, National Institute of Technology Nagaland, Chumukedima, Nagaland 797103 India

**Keywords:** Nanoscale devices, Nanoparticles, Two-dimensional materials

## Abstract

NiO nanoparticles (NPs) synthesized using glancing angle deposition (GLAD) technique over MgZnO thin film was used to design a novel memory device. The NiO NPs with average diameter ~ 9.5 nm was uniformly distributed over the MgZnO thin film surface. The MgZnO thin film/NiO NPs memory device when measured for the C-V hysteresis characteristics at varying sweep voltage demonstrated a charge trapping and de-trapping mechanism. Moreover, the device exhibited low interface states density (D_it_) (1.45 × 10^10^ eV^− 1^ cm^− 2^) at 1 MHz and large capacitive memory of  ~ 6 V at ± 7 V. The large memory window was attributed to the better interface quality between MgZnO thin film and NiO NPs. Additionally, the device also exhibited good endurance over 1000 programme/erase cycles and longer time charge retention up to 2 × 10^4^ s. The improved performance of device and more charge accumulation capacity was primarily due to the large effective area and quantum confinement effect owing to NiO NPs. Further, on performing a resistive switching analysis, the device could show a good on-off ratio (R_HRS_/R_LRS_) of 1.24 × 10^2^. Therefore, the proposed device structure can be a good option for future memory applications.

## Introduction

The ability of nanoparticle (NP) based memory application to exhibit high non-volatile features, such as greater endurance, retention, and scalability, has garnered a lot of interest in recent reported literatures^1–3^. Even though non-volatile memory (NVM) has advanced significantly, they might not be the best option to fulfil the changing requirements for storage. The main disadvantage is an inability to reduce storage cells and long-time data retention which impending the sustainable development of exploding data^4^. In order to overcome theses issues, the nanoscale device gives the quantum confinement effect and Coulomb blockade effect which boost the long-time data retention capability and high charge storage with improved uniformity control^5^. A novel route for the fabrication of two terminal memory devices is engraved by a variety of nanomaterials, including metal oxide thin film (TF)^6^, nanowires (NWs)^7,8^ and nanoparticles (NPs)^9^. Among, various nano-dimensions, the combination of film decorated with NPs hybrid structure can be a promising candidate for enhanced storage device in terms of capacitive memory. Due to the stronger electric field produced in the small geometrical structure, they have the potential to be used in the production of memory and data storage devices. Metal-oxide-semiconductor (MOS) based memory device has drawn a lot of interest due to its diverse application, high retention, low power consumption, and high data storage density.

Mo et al.^10^ studied 3D high-density capacitive memory application of metal/ferroelectric (FE)-HfO_2_/IGZO/metal structure showed high endurance and retention property upto 10^8^ program/erase cycles. Lahiri et al.^7^ reported Er-doped TiO_2_ NWs MOS capacitive memory with low interface state density (~8.72 × 10^10^ eV^− 1^ cm^− 2^) and improved memory window of ~3.52 V at ± 10 V. Similarly, Ta_2_O_5_ TF synthesized using electron-beam evaporation technique reported by singh et al.^11^ showed enhanced capacitive memory of 7.9 V at ± 10 V and stable resistive switching behaviour with resistance ratio of 10^2^. Among several MOS, zinc oxide (ZnO) holds unique characteristics such as wide bandgap (3.37 eV), strong exciton binding energy (60 meV)^6^, and tuneable dielectric constant^12^. Moreover, zinc interstitial defects and oxygen vacancies are the two main defect sites in ZnO which forms recombination centers under the influence of electric field. This is due to the fact that the relative trapping sites at an oxygen vacancy in ZnO are significantly smaller than those of zinc vacancy^13^. Therefore, minimizing oxygen-related defects essentially improve the charge storage performance can be achieved by doping with the material which possess minimum lattice mismatch and similar ionic radii. Since Zn^2+^ (0.060 nm) and Mg^2+^ (0.057 nm) have ionic radii comparable to those of other materials^14^, replacing Zn with Mg would not cause significant lattice deformation or phase change, making Mg an appropriate dopant element for device application. However, thinner oxide layer causes tunnelling effect is the source of the high leakage current, which lowers the charge carrier trap density and also cause degradation in retention performance of the memory device^15^. To boost the charge storage capacity, several authors have modified the surface functionalization using the NPs at the top of nanostructure. NPs based memory device drawn an attention due the drastic reduction of leakage current as compare to continuous floating layer in conventional memory device^16–19^. NP-equipped memory devices avoid the effect of continuous floating layer due the presence of discrete NPs layer which attributed an improved performance over state-of-the-art memory device, with superior charge storage capacity, larger data retention times and decreased power consumption^15,16^. Jeff et al.^20^ demonstrated the Pt NPs memory device fabricated using atomic layer deposition technique obtained enhanced memory window of 6.5 V and superior retention time of 10^5^ s. Park et al.^21^ reported the Au NPs embedded MOS capacitance chrematistics stored large number of charge carriers and low decay of capacitance about 2% after 10^4^ s. Au NPs decorated TiO_2_ NWs based capacitive memory was investigated by Kashyap et al.^22^ showed enhanced capacitive memory window of 12.65 V and high charge trap density of 10^14^ cm^2^. Moreover, several authors also studied the metal oxide based memristors devices such as Carlos et al.^3^ showed bipolar resistive switching behaviour with retention time of 10^5^ s and good endurance characteristics. Similarly, Moirangthem et al.^33^ and Laishram et al.^34^ also demonstrated resistive switching with resistance ratio of ~252 and ~13 respectively.

Despite the fact that metal NPs have a variety of options for nano-floating gate memory, there are some restriction such as high leakage current, possibilities of oxide formation when exposed in environment over longer time period and challenging to control the size of NPs which prevent the enhancement of capacitive memory parameters. In order to overcome these issues metal-oxide NPs could be a better option which possess typically high dielectric constant [Robertson 2004], stable under the varying environmental condition^23^, exhibit low leakage current^24^, effective charge trapping capability^25^, and controlled synthesis at nanoscale^26,27^. Furthermore, MgZnO TF and NiO NPs form a type-II junction interface which ensure effective charge accumulation^28^, low interface state density and large memory window. These can be achieved by controlled deposition of NiO NPs at low-temperature RF sputtered GLAD technique which prevents the interfacial reaction and reduce the occurrence of defect sites.

In this study authors have investigated the NiO NPs decorated MgZnO TF based capacitive memory application using RF magnetron sputtering incorporated glancing angle deposition (GLAD) technique to obtain controlled growth of nanostructure. Owing above comprehensive study of recently reported literature there is no existing article on MgZnO TF/NiO NPs based capacitive memory device. The MgZnO TF/NiO NPs sample was examined their structural and crystalline phases using field-emission gun scanning electron microscopy (FEG-SEM) and X-Ray Diffractometer (XRD). The memory characteristics of NiO NPs assisted MgZnO TF device was analysed using capacitance (C) - voltage (V) hysteresis curve at 1 MHz over varying sweep voltage from ± 1 to ± 7 V. The C-V and conductance (G)- voltage (V) characteristics were conducted at various frequency range from 100 kHz to 4 MHz which showed the charge trapping capability of NiO NPs decorated MgZnO TF device. Moreover, device also exhibited significant improvement in endurance and retention properties over the large number of programme (P)/erase (E) cycles, which holds potential for better memory device application. Moreover, the MgZnO TF/NiO NPs device was also analysed for resistive switching behaviour at room temperature showed high resistance ratio (R_HRS_/R_LRS_) which implies the potential resistive memory application for next generation storage device.

## Experimental procedure

### Fabrication of NiO NPs decorated MgZnO TF

**Fig. 1:**
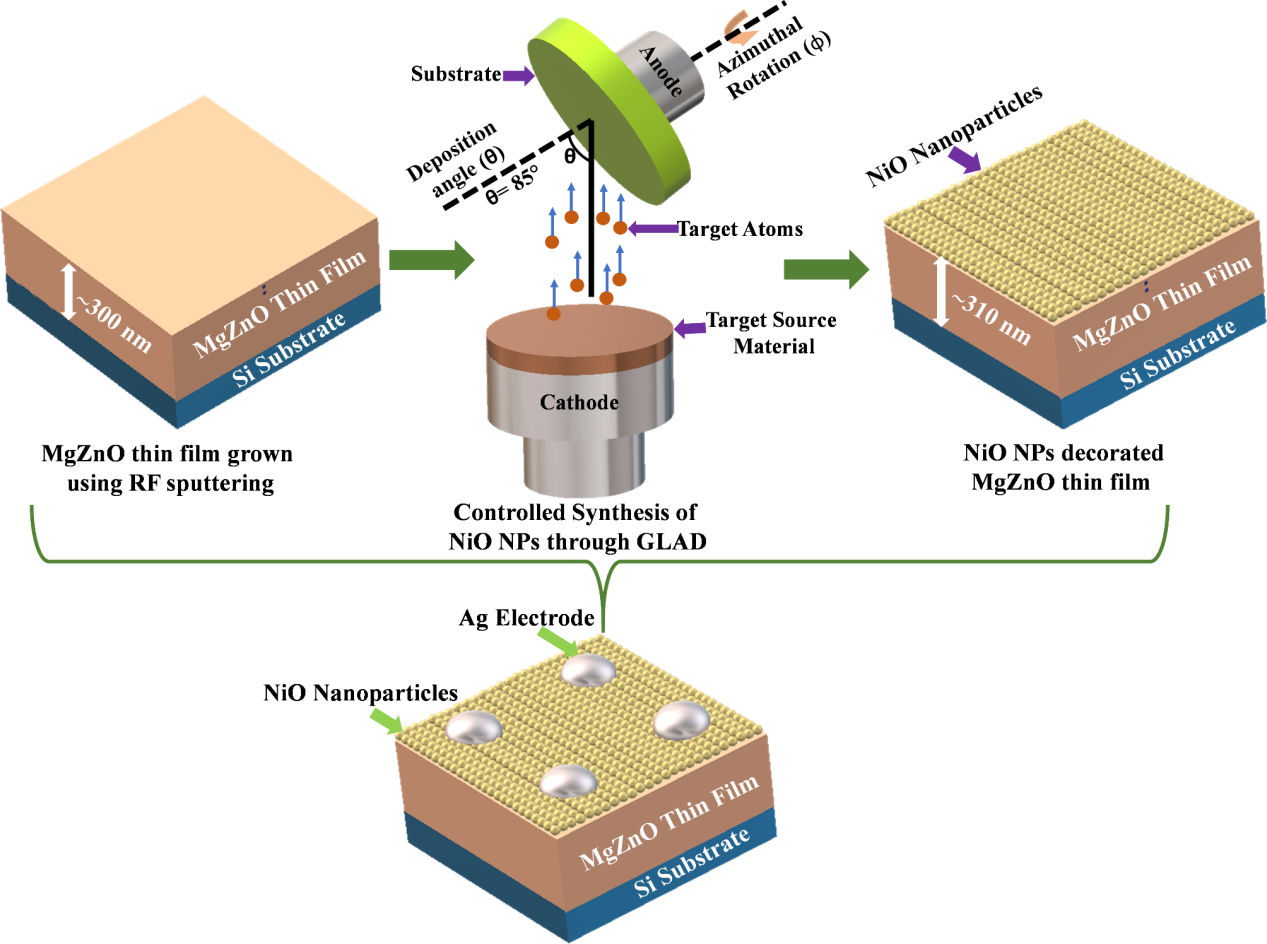
Schematic of the controlled growth of NiO nanoparticles (NPs) decorated MgZnO thin film (TF) and complete device structure.

NiO NPs decorated MgZnO TF was fabricated on n-type silicon (Si) substrate of dimension 1 cm × 1 cm using RF magnetron sputtering method (Smart coat 3.0, HHV INDIA) as illustrated in Fig. 1. Before deposition, the n-type Si substrate was cleaned using an ultrasonicator for five minutes in each of the following solutions: acetone, methanol, and deionized (DI) water to ensure the surface is free from contamination. The entire deposition was conducted in high vacuum environment. To generate a high vacuum environment (4 × 10^− 6^ mbar), the chamber was evacuated to reduce the presence of gas molecules that interfere with the sputtering process. The high purity Mg_0.1_Zn_0.9_O (99.99%) sputtering target was used to grow a TF of thickness ~ 300 nm on Si-substrate. In order to achieve uniform film thickness, the substrate was continuously rotated at 70 revolution per minute (RPM). On the top of MgZnO TF surface the NiO NPs of ~ 10 nm were decorated (high purity NiO (99.99%) sputtering target). The deposition of NiO NPs were accomplished using the glancing angle deposition (GLAD) technique, in which the substrate was placed at an angle of 85° with respect to the source target inside the deposition chamber. Since it doesn’t require a high temperature or catalyst to develop the nanostructure, the GLAD technique is recognized as the simplest method for fabricating controlled shape and in-plane aligned NPs. Several advantages such as self-alignment due to the shadowing effect, porosity of the film can be controlled by simply changing the deposition angle, and ability to produce heterogeneous nanostructures in precisely makes the GLAD technique promising for various nano-dimensional structure. The entire deposition was carried out using RF power supply of 150 W, argon (Ar) as a sputtered gas with the flow rate of 12 sccm (standard cubic centimetre per minute) and at high constant deposition pressure of 1.5 × 10^− 3^ mbar. The rate of deposition was maintained at 0.5 Å/s during the whole process and monitored using digital thickness meter (DTM). Furthermore, a circular Ag electrode of diameter 1.19 mm was grown on the top of MgZnO TF/NiO NPs of thickness ~ 30 nm by using shadow masking technique. The deposition of Ag metal electrode was also carried out under high vacuum condition using DC magnetron sputtering technique with power of 50 W, voltage 400 V, current 0.5 A and constant deposition pressure of 1.5 × 10^− 3^ mbar respectively. Indium (In) metal was used as the bottom electrode while measuring the electrical characteristics.

### Characterization

The fabricated sample was characterised structurally and morphologically using field-emission gun scanning electron microscopy (FEG-SEM) (Zeiss, Ultra 55) including energy dispersive X-ray spectroscopy (EDS) and X-Ray Diffractometer (XRD) with Cu Kα radiation (λ~ 1.54 Å) (Rigaku Ultima IV) respectively. UV-vis-NIR spectrophotometer (Hitachi UH4150), was used to investigate the optical property of the sample. The electrical characteristic (current (I) – voltage (V), capacitance (C) - voltage (V)) of fabricated device was analysed using semiconductor characterization system (Keithley 4200-SCS).

## Result and discussion

### Structural, morphological and optical analysis

**Fig. 2:**
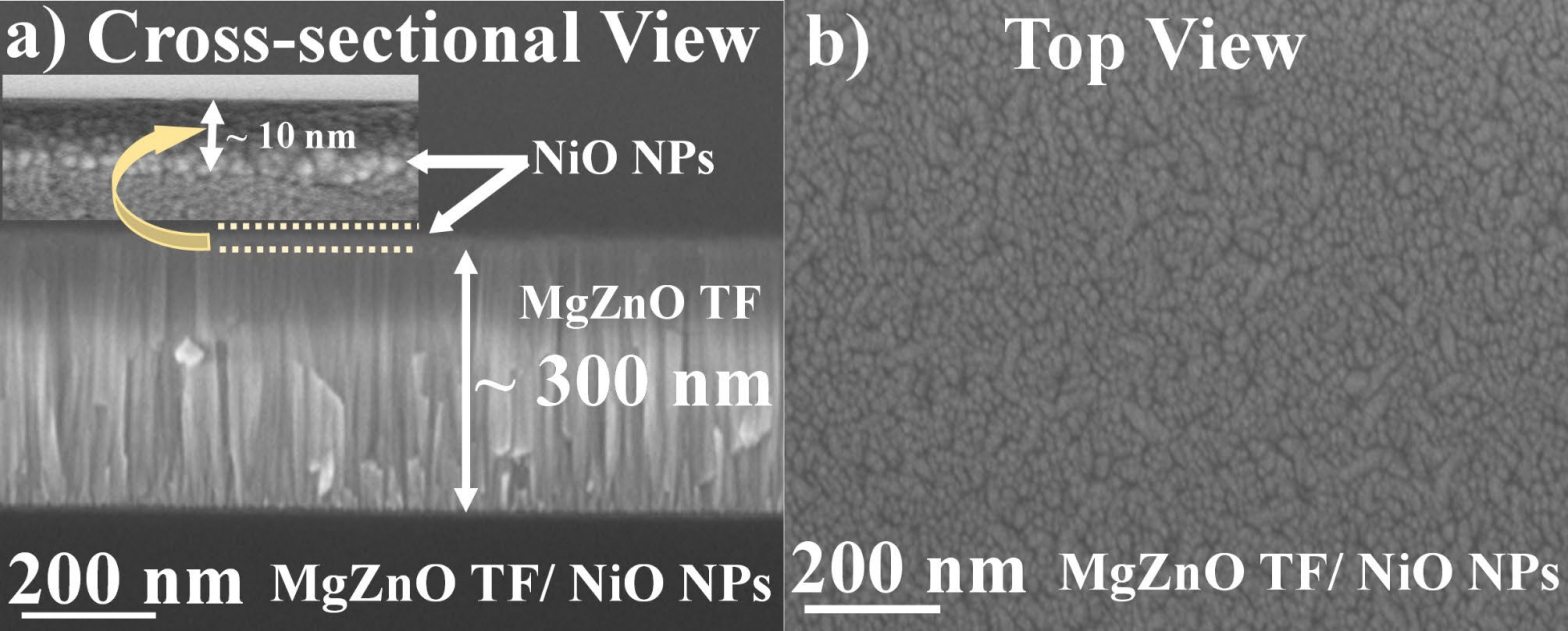
FE-SEM image of NiO NPs decorated MgZnO TF (**a**) Cross-sectional view (magnified image of NiO NPs inset), (**b**) top view, (**c**) statistical data of the average size of NiO NPs, (**d**) surface plot of NiO NPs, and (**e**) EDS spectrum of MgZnO TF/NiO NPs.

Figure 2(a) – (e) illustrate the surface morphology of the fabricated sample which has been studied using FE-SEM analysis. The cross-sectional and top view of MgZnO TF/NiO NPs sample is shown in Fig. 2 (a) and (b) respectively. The overall thickness of MgZnO TF/NiO NPs was found to be ~ 310 nm (MgZnO TF ~ 300 nm and NiO NPs ~ 10 nm). The magnified image of NiO NPs cross-sectional view shown in Fig. 2a (inset). From top view it can be observed that the NPs grown uniformly through out the surface of MgZnO TF. Based on Fig. 2 (b), the NPs size histogram and surface plot of top view (Fig. 2 (c) and (d)) were analysed using imageJ software and it was observed that the wider particle size distribution. The average size of decorated NiO NPs was calculated to be ~ 9.5 nm with size varying from 3 nm to 20 nm as shown in Fig. 2 (c). Furthermore, the EDS spectrum is depicted in Fig. 2 (e), where the elements oxygen (O_2_) (39.31%), magnesium (Mg) (3.43%), silicon (Si) (48.93%), nickel (Ni) (0.15%), and zinc (Zn) (8.18%) are successfully detected.

**Fig. 3:**
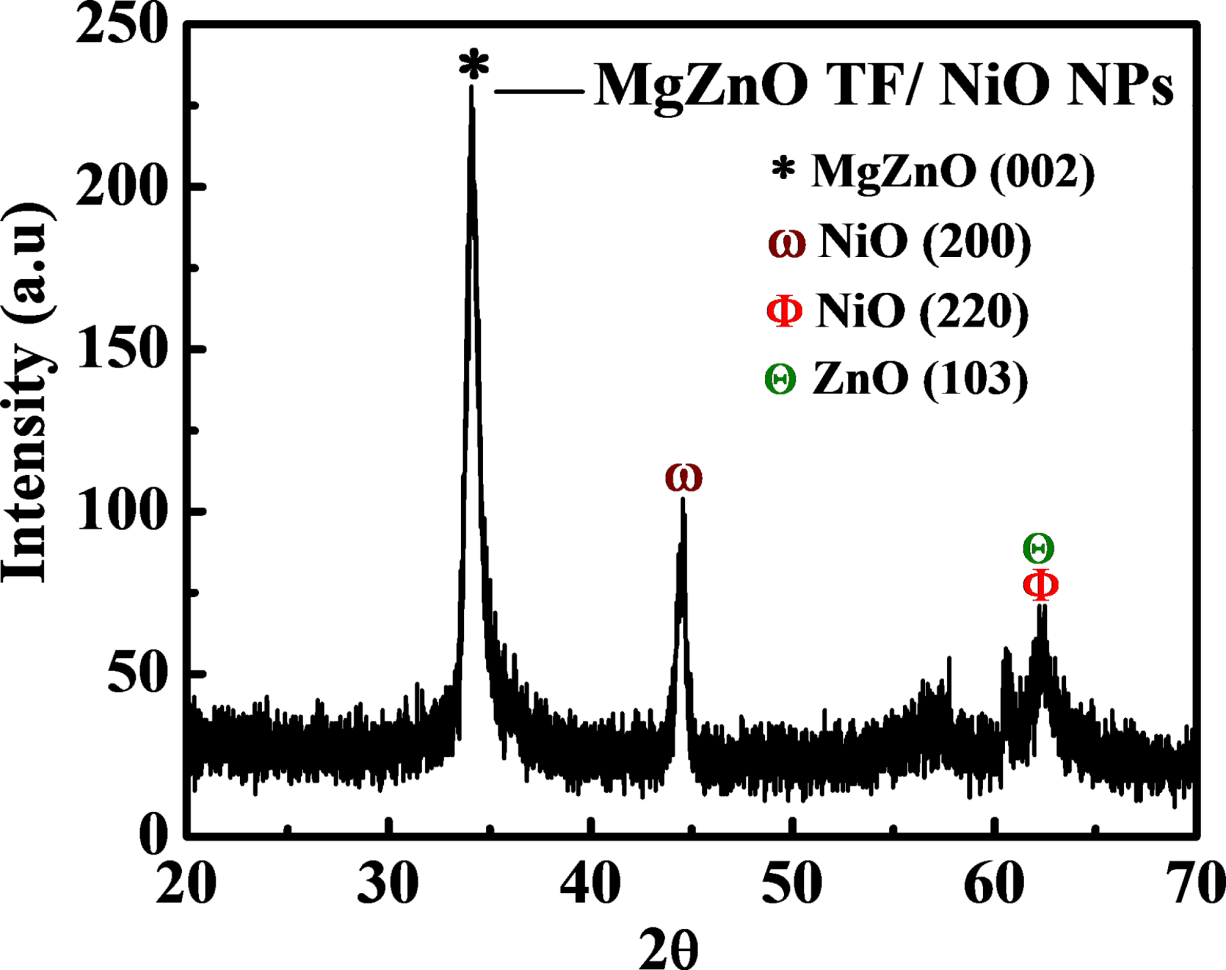
XRD spectrum of NiO NPs decorated MgZnO TF.

The XRD analysis as depicted in Fig. 3 was performed in order to obtain the atomic arrangement and crystallographic information of the fabricated sample at room temperature. A sharp peak at around 34.08° observed, corresponding to the c-axis orientated (002) plane which also confirms the hexagonal wurtzite structure of MgZnO^14^. In addition, peaks at 44.53° corresponds to the (200) plane of NiO and also obtained doublet peaks at 62.31° indicating ZnO (103) and NiO (220) respectively^29,30^.

**Table 1:** Structural parameters of MgZnO TF/NiO NPs crystallite size (D), dislocation density (δ), and Micro strain (ε) estimated by XRD data and average particles size from FE-SEM data.

Sample	Crystallite size, D (nm)	Dislocation density, δ (×10^− 2^)	Micro strain, ε (×10^− 3^)	FE-SEM data average NPs size (nm)
MgZnO TF/NiO NPs	10.5	0.8	11.7	9.5

The average crystallite of MgZnO was estimated using Debye-Scherrer Eq. (1)^30^.

1 $$\:\:\:\:D=\frac{0.9\:\lambda\:}{\beta\:\:cos\theta\:}$$

Where D is the crystallite size, λ indicates the wavelength of X-ray radiation (1.54 Å), β is the full-width half maxima (FWHM) in radian and θ is the angle of diffraction. The estimated D value was found to be 10.5 nm. Moreover, the dislocation density (δ) and macro strain (ε) were also calculated using the Eqs. (2) and (3)^14^.

2 $$\:\delta\:=\:\frac{1}{{D}^{2}}$$

3 $$\:\epsilon\:=\:\frac{\beta\:}{4\:tan\theta\:}$$

**Fig. 4:**
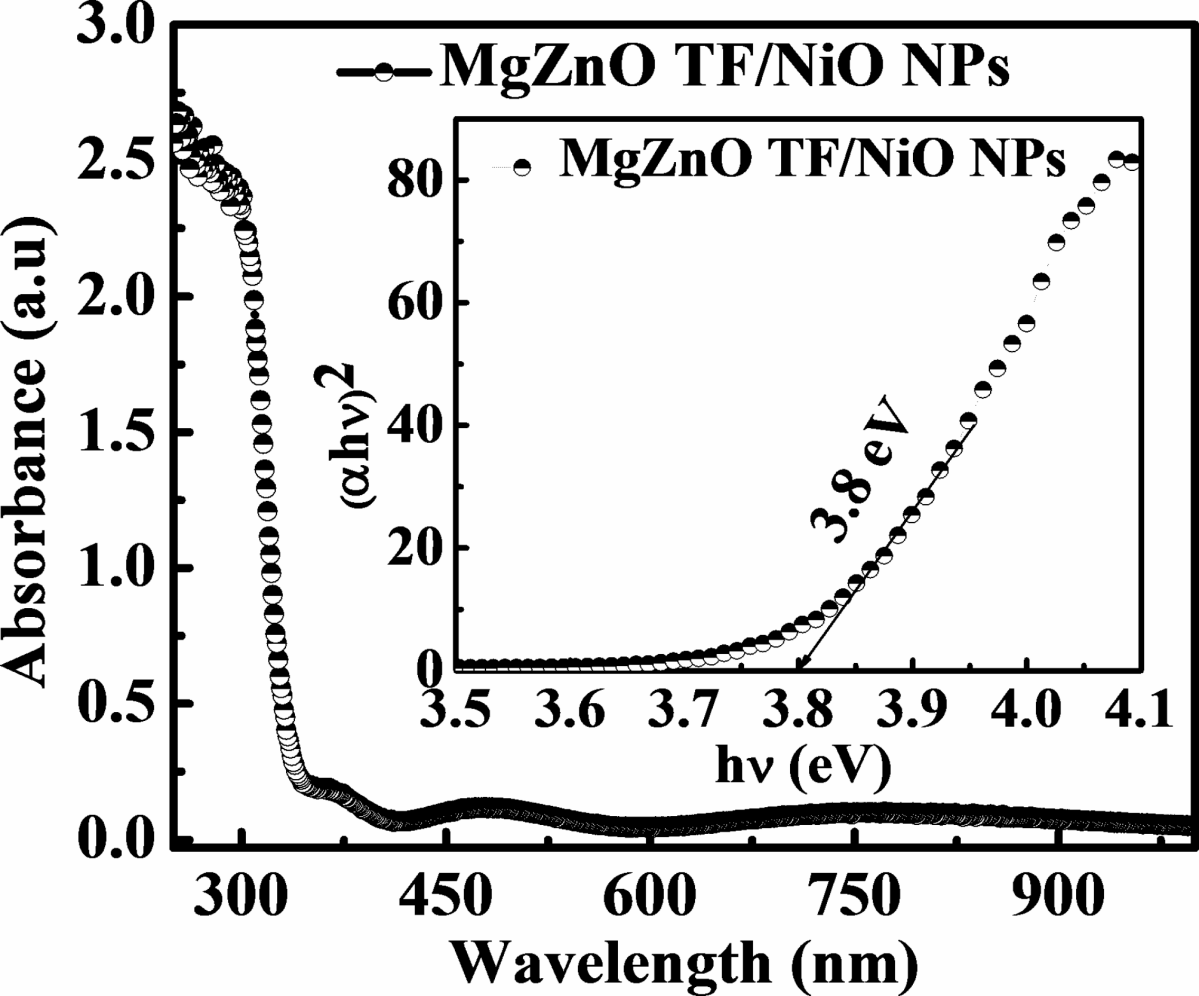
Optical absorption of NiO NPs decorated MgZnO TF and corresponding Tauc plot (inset).

The estimated values of δ and ε were found to be 0.8 and 11.7 for the MgZnO TF/NiO NPs sample (shown in Table 1). Figure 4 depicts the UV-visible spectra of the NiO NPs decorated MgZnO TF ranging from 250 nm to 1050 nm was conducted at room temperature. The sample showed strong absorption in the UV region. The primary optical absorption may be attributed to the MgZnO TF since the thickness of MgZnO TF (~300 nm) was relatively larger than the NiO NPs (~9.5 nm). The higher absorption was observed in UV region due to the wide bandgap nature of MgZnO and NiO material. Moreover, the corresponding bandgap of MgZnO TF/NiO NPs sample was found to be 3.8 eV as plotted in Fig. 4 (inset) which was estimated using Tauc’s Eq. (4)^6^.

4 $$(\upalpha {\rm hv})={\rm A(hv}-E_{\rm g})^{1/2}$$

### Capacitance (C)–voltage (V) and conductance (G)–voltage (V) characteristics

**Fig. 5:**
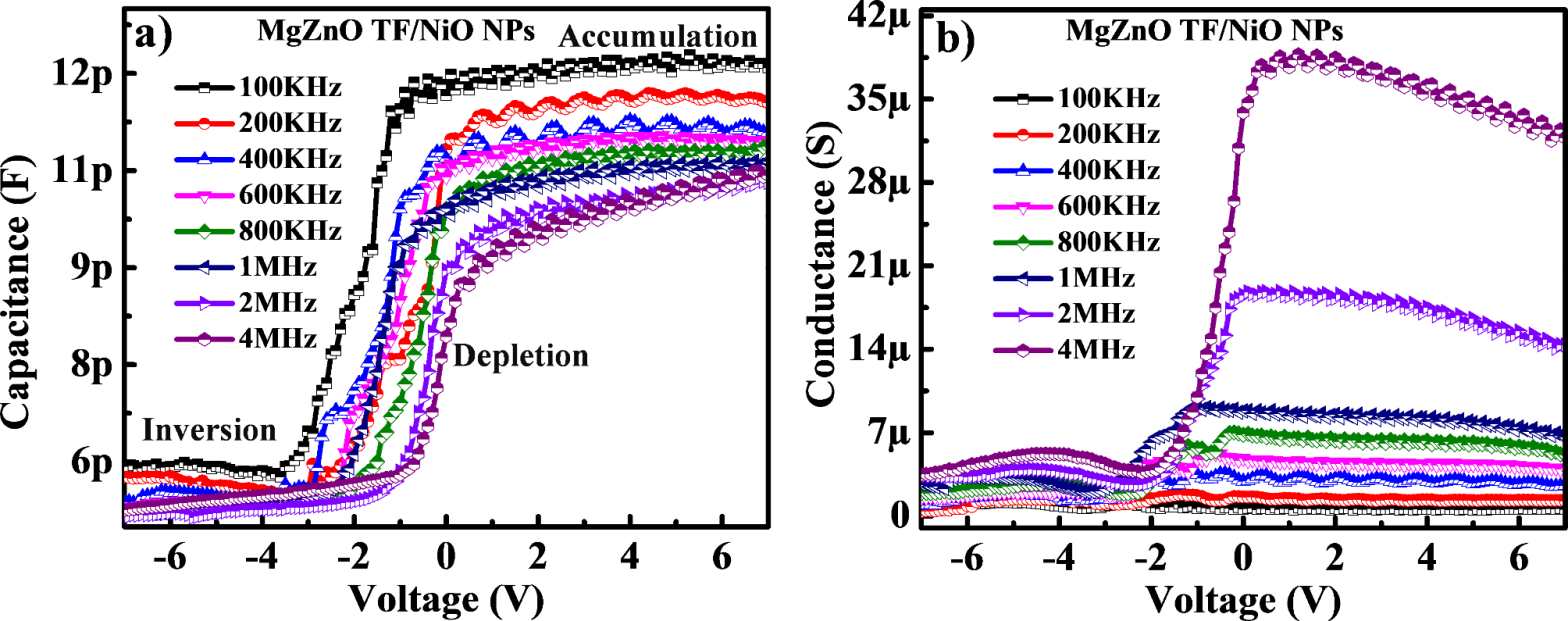
(**a**) Capacitance (C)–voltage (V) and (**b**) conductance (G)–voltage (V) characteristics of MgZnO TF/NiO NPs device.

Figure 5 (a) and (b) shows the measured capacitance (C) and conductance (G) plot versus applied voltages (V) with varying frequency range from 100 kHz − 4 MHz to comprehend the charge trap density in the MgZnO TF/NiO NPs device. The C-V characteristics shown in Fig. 5 (a) confirms the typical n-type metal oxide semiconductor (MOS) capacitor which clearly displays three distinct regions as accumulation, depletion, and inversion region. The strong accumulation capacitance was observed at + 7 V for the lower frequency and vice-versa this may be due to effect of series resistance and localised interface state at the Si/MgZnO TF^6^. Under a positive gate bias, it exhibits a high capacitance in the accumulation region and a low capacitance in the inversion region at negative bias. The ac response associated with interface traps and free carriers are the cause of the frequency dispersion in the C-V and G-V curve^31^. The capacitance in accumulation region exhibits good response towards the change in frequencies. As the frequency rises from 100 kHz to 4 MHz, the capacitance value tends to decreases from 1.23 × 10^− 11^ F to 1.01 × 10^− 11^ F indicating the presence of deep states with a long time constant^6,31^. The yield of higher capacitance at low frequency attributed due to the trapped electrons. The capture and emission time constants of the states determine this trapping and de-trapping mechanism which is administered by density states and carriers’ dynamics. Moreover, the interface states responsiveness decreases with increasing AC signal frequency, this occur primarily due to at lower frequency the interface states effectively follow the slow change in AC signal which contributes to high capacitance value. Whereas, at higher frequency the AC signal changes too quick that the interface states could not be able to follow.

Furthermore, the G-V curve shown in Fig. 5 (b) it is observed that the maximum G value was found at higher frequency and as frequency decreases the G value also decreases. This may be due the effect of series resistance (R_s_) and interface states density (D_it_). Thus, the D_it_ and R_s_ was estimated from G-V curve using Eqs. (5) and (6) respectively^6^.

5 $$\:{D}_{it}=\frac{\left(\frac{{G}_{max}}{\omega\:}\right)\left(\frac{2}{qA}\right)}{{\left(\frac{{G}_{max}}{\omega\:{C}_{ox}}\right)}^{2}{+\:\left(1-\frac{{C}_{m}}{{C}_{ox}}\right)}^{2}}$$

6 $$\:{R}_{s}=\frac{{G}_{ma}}{{\left({G}_{ma}\right)}^{2}+{\left(\omega\:{C}_{ma}\right)}^{2}}$$

Where, C_ox_ is the capacitance in accumulation region, A is the area of device, ω denotes the angular frequency, q is electronic charge, C_m_ is the maximum capacitance corresponding to G_max_, G_ma_ and C_ma_ are the measured value of capacitance and conductance at strong accumulation region.

**Fig. 6:**
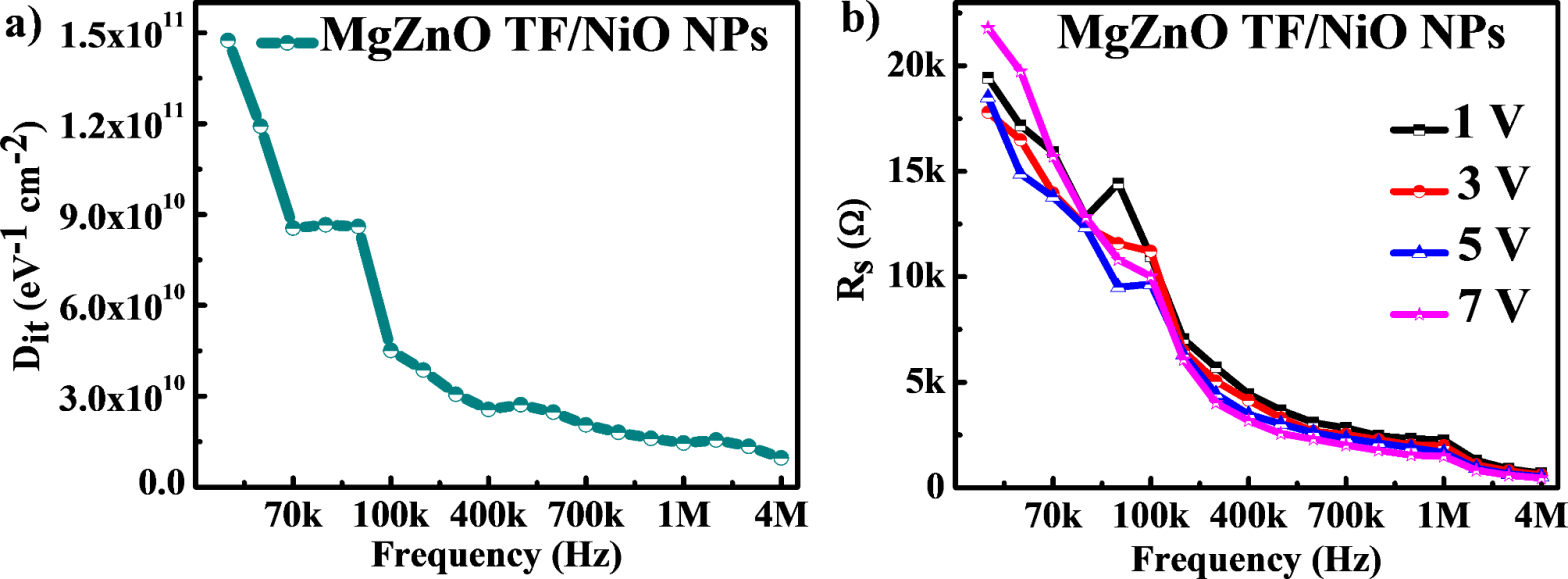
(**a**) Interface trap density (D_it_) and (**b**) series resistance (R_s_) of MgZnO TF/NiO NPs device.

The computed D_it_ and R_s_ versus frequency is depicted in Fig. 6 (a) and (b) respectively. The value of D_it_ found to be decreasing as the frequency increases. This suggests that the alternating current at the higher frequency is not followed by the interface state carriers^7,32^. Authors have also reported similar behaviour of D_it_ at higher frequency in the recent article^6^. The value of D_it_ was found to be 1.45 × 10^10^ eV^− 1^ cm^− 2^ at 1 MHz, which is better then the recently reported literatures^11,33,34^. The low D_it_ value mainly due to defect free interface, controlled growth of NPs, low lattice mismatch between MgZnO TF and NiO NPs, and low-temperature fabrication technique (like RF sputtering). Moreover, the R_s_ versus frequency is shown in Fig. 6 (b), it can be observed that the calculated value of R_s_ declined as frequency increased and remains constant at sufficiently higher frequency. This may be due to a higher frequency, the trapped charges at interface could not follow the ac signal^6,35^. In addition, a change in R_s_ was found to be consistent in the accumulation region at higher frequencies but the variation is noticed especially in inversion and depletion regions. This demonstrates the effective R_s_ value in the accumulation region at high frequency of the MgZnO TF/ NiO NPs device. Moreover, the NiO NPs-decorated device exhibited high resistance to carrier movement and lower conductance, indicating charge trapping at the MgZnO TF/NiO NPs interface.

### Capacitive memory characteristics of NiO NPs decorated MgZnO TF device

**Fig. 7:**
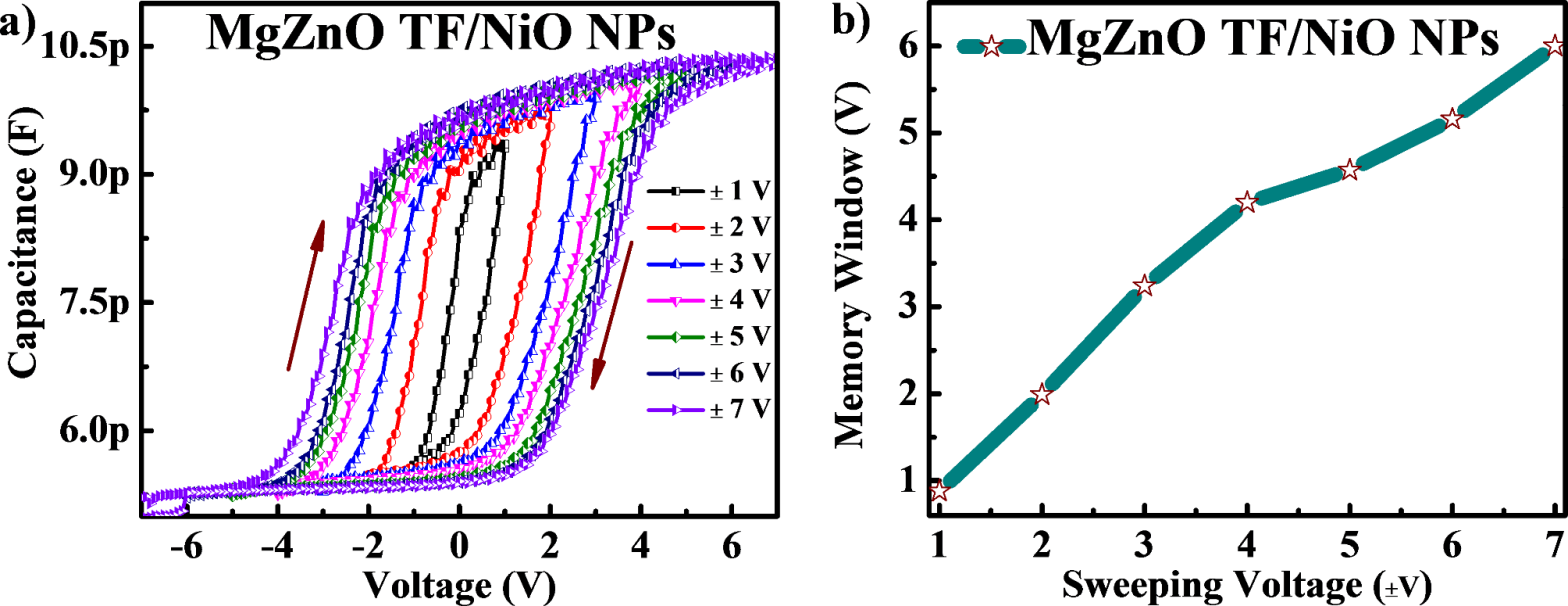
(**a**) C-V hysteresis at varying sweep voltage (± 1 V to ± 7 V) and (**b**) memory window Vs sweeping voltage of NiO NPs decorated MgZnO TF device.

Figure 7 (a) depicts the C − V hysteresis curve of the NiO NPs-decorated MgZnO TF at a frequency of 1 MHz with varying sweeping voltages from ± 1 to ± 7 V to study the charge retention property of the device. The device showed clockwise C-V hysteresis loops, which demonstrate that the device charging and discharging by sweep voltages from the inversion region to the accumulation region and swept back to the inversion region. It can be observed that the flat band voltage (V_FB_) shifted in the direction of more positive voltage when the bias voltage was swept from the inversion to accumulation region. This is most likely due to the existence of NiO NPs and the interface states that exist between NiO NPs and MgZnO TF. The V_FB_ shift to the positive side also suggests that most of the electrons are trapped might be due the presence of oxygen vacancies^6^. In addition, the V_FB_ shift also owing the quantum confinement effect of NiO NPs. The resulting C − V hysteresis was dependant on the voltage sweeping range and increases with the rise in bias voltage indicating that more charges are injected into NiO NPs positioned in the accumulation area. Moreover, the device capacity to store charge was shown by the memory window, also known as the threshold voltage shift in the gate voltage bi-sweeps (± 1 to ± 7 V). Figure 7 (b) shows the memory window (MW) under varying sweep voltage from ± 1 to ± 7 V. The MW of MgZnO TF/ NiO NPs device was found to be 0.88 V, 3.42 V, 4.57 V and 6 V under the varying sweep voltage at ± 1 V, ± 3 V, ± 5 V and ± 7 V respectively. The NiO NPs have a high surface-to-volume ratio, leading to a larger charge accumulation region. This enhances charge storage at the interface, thereby increasing the device’s capacitance. The obtained MW and low D_it_ value were found to be improved as compare to recently reported literatures^6,11,33,34,38^. Therefore, the significant enhancement in MW was observed mainly due to efficient charge trapping in the NiO NPs and MgZnO TF/NiO NPs interface, which serve as a charge trapping multilayer interface.

**Fig. 8:**
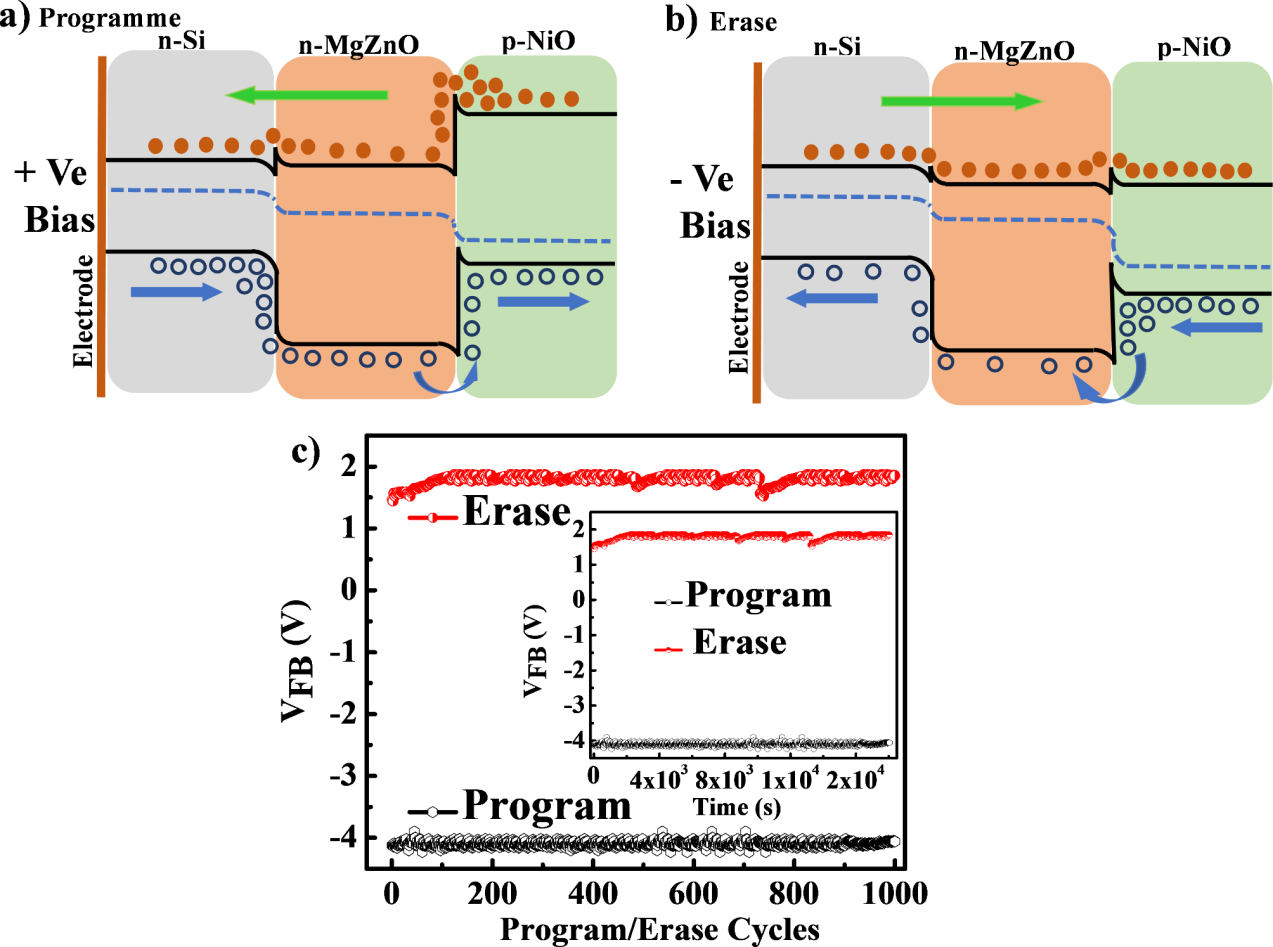
Band structure showing (**a**) Programme, (**b**) Erase, and (**c**) Endurance and retention (inset) of NiO NPs decorated MgZnO TF device.

Figure 8 (a) and (b) shows the band diagram of programme and erase (P/E) mechanism of MgZnO TF/NiO NPs device. In this case the programme cycle starts with forward sweep voltage from − 7 V to + 7 V and subsequently the reverse sweep voltage + 7 V to -7 V gives the erase cycle. The device is more forward bias when the − 7 V sweep voltage connected at the rear contact of n-Si, which allow the easy passage of charge carrier to flow from MgZnO TF to NiO NPs and reaches to Ag electrode that reduces the capacitance value of the device. In forward bias condition the holes were trapped between the interface of MgZnO TF and NiO NPs, while electrons were easily injected from the substrate. Moreover, it has been reported previously^6^ that the MgZnO contains higher oxygen vacancies and deep level trap states which act as a shallow trap level to introduce larger energy levels into the MgZnO TF. Thus, as a result the forward bias voltage increases the greater number of holes trapped in NiO NPs, which allow holes to leak through trap assisted tunnelling during the programme cycle. The device showed small variation in its capacitance value when the forward bias voltage changes from − 7 V to -4 V which is termed as depletion region. The device lost its forward bias condition when the voltage was raised from − 4 V to 0 V, attributes higher barrier height at MgZnO TF and NiO NPs interface which restricts the flow of electron. The device demonstrated a rise in reverse bias when the voltage was scanned from 0 V to 7 V showed almost constant capacitance value referred as accumulation region. Furthermore, the programme erase occurs during the reverse voltage scan from + 7 V to -7 V. The device exhibits a greater reverse bias condition and a large accumulation capacitance at + 7 V. When the voltage scan begins at + 7 V and proceeds to 0 V, the reverse bias decreases and the accumulated charge carriers begin to move in the junction due to a decrease in the barrier height consequently the current conduction increases. As a result, the device experiences an erase cycle because the accumulated charge carriers flushed out rapidly and collected by the electrode which reduces the capacitance value. Further, the voltage scanned from 0 V to -7 V the device remains in forward bias condition which showed lower capacitance value. The P/E cycles provides the existence of a capacitive MW in terms of V_FB_ difference in the device. The MgZnO TF/NiO NPs device showed the MW of ~6 V by rapid change of its capacitance from P/E mode.

Furthermore, in order to understand the reliability and memory performance of MgZnO TF/NiO NPs device was examined for endurance and retention at room temperature as shown in Fig. 8 (c). The endurance property of the device was investigated by varying bias voltage − 7 V to + 7 V for 1000 cycles of P/E. For 1000 P/E cycles, the V_FB_ difference between programme and erase was almost the same, indicating that the device has high stability and endurance characteristics. Retention is another crucial parameter for memory device to determine the time up to which the memory property can be retained by the device. The retention property of device was measured as shown in Fig. 8 (c) inset by varying sweep voltage from − 7 V to + 7 V for longer time period of 2 × 10^4^ s. It was observed that the MW remains constant up to 2 × 10^4^ s, which signifies that the device possesses good retention characteristic and can hold potential candidate for memory application.

### Resistive switching characteristics of NiO NPs decorated MgZnO TF device

**Fig. 9:**
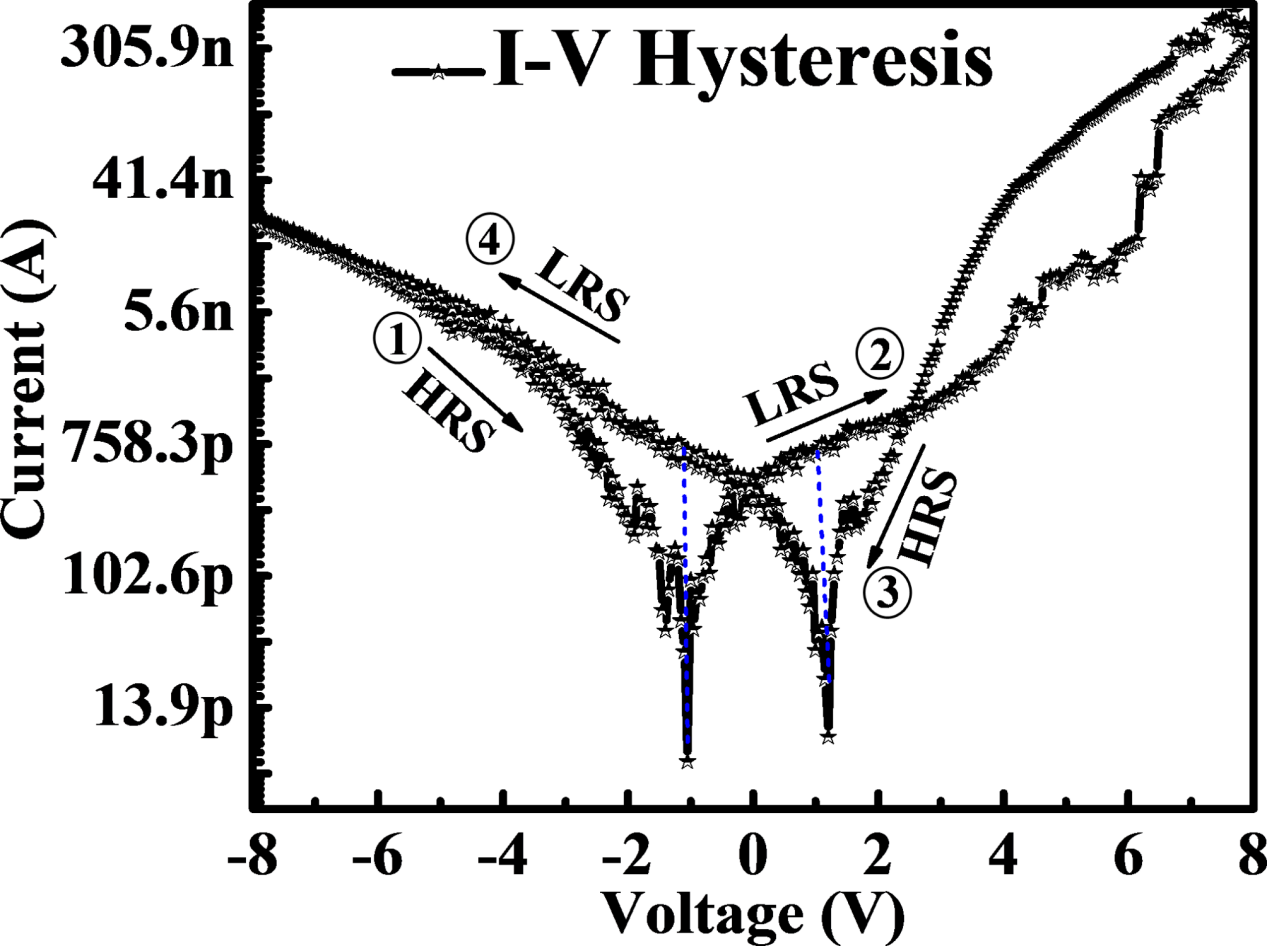
Current (I)-voltage (V) hysteresis semi log curve of MgZnO TF/NiO NPs device at room temperature.

Figure 9 demonstrate the phenomena of resistive switching of NiO NPs decorated MgZnO TF using a semi-log scale at room temperature under dark condition. The 500 consecutive switching cycles were measured at varying bias voltage from − 8 V to + 8 V sweep in the order of -8 V → 0 V (Path 1), 0 V → +8 V (Path 2), + 8 V → 0 V (Path 3) and 0 V → -8 V (Path 4) respectively. To prevent the device’s catastrophic electrical breakdown during the measurements, the compliance current was set at 0.1 A. The bottom electrode was grounded, while the top electrode (Ag) received the supply voltage (-8 V to + 8 V). The hysteresis shows significant variation in low resistance state (LRS) SET mode and high resistance state (HRS) RESET mode by the variation of current value of the device. The SET and RESET operations consecutively examined the HRS and LRS after the forming process had triggered the device. The primary source of resistive switching in MgZnO TF/NiO NPs device is the creation or rupture of conductive filaments which occur mainly due to electrochemical metallisation mechanism and oxygen related vacancies^36^. As the top electrode (Ag) is positively biased, NiO NPs get ionized in close proximity to the electrode. Subsequently, the NiO NPs might offer moveable Ni^2+^ ions and diffuse into the negatively charged bottom electrode of the MgZnO TF, which reduces the NiO atoms that involves in creating the conducting filament. Such conducting routes could be broken during the RESET process by the joule heating action under the opposite electric field polarity^37^. It is observed that the device remained at LRS at higher bias voltages of ± 8 V and as bias voltage changes from ± 8 V → 0 V the device went at HRS. For the voltage scan from − 8 V to + 8 V the device showed first HRS at -1.02 V with resistance of 0.17 × 10^12^ Ω and first LRS at + 1.21 V with resistance 0.14 × 10^10^ Ω respectively. Similarly, while scan from + 8 V to -8 V the device second HRS at + 1.21 V with resistance 0.13 × 10^12^ Ω and second LRS at -1.02 V with resistance 0.13 × 10^10^ Ω respectively. By generating a significant resistance change between the LRS and HRS regions, the hysteresis curve demonstrates the good resistive switching property. Thus, the ratio of R_HRS_/R_LRS_ at -1.02 V and + 1.21 V are found to be 1.24 × 10^2^ and 0.91 × 10^2^ respectively which shows the potential for resistive memory application and can be further explored for analysing its various parameters. Table 2 presents the comprehensive comparison of recently reported literatures.

**Table 2:** Comprehensive performance index of recently reported memory devices.

Device Structure	Memory window (V)	D_it_ (eV^-1^ cm^-2^)	Resistance ratio	Ref.
Ag NPs/HfO_2_ TF	2.21	8.81 × 10^11^	~252	^33^
p-Si/MgZnO TF	1.8	2.0 × 10^10^	-	^6^
Axial NiO-NW/β-Ga2O3 NW HS	2.83	1.13 × 10^11^	-	^38^
TiO_2_ NWs	1.67	1.99 × 10^12^	-	^22^
Au NPs/GO	0.9	-	-	^17^
Au/SiO_x_/p-Si	1.76	2.65 × 10^11^	13	^34^
Ag NPs/ZnO	1.5	-	-	^9^
WO_3_ NW	5.96	4.54 × 10^10^	-	^39^
Au/TiO_2_ NW/GO TF	3.72	1.12 × 10^13^	-	^19^
Au/Ta_2_O_5_ TF	7.9	2.47 × 10^11^	~10^2^	^11^
n-Si/In_2_O_3_ NW/Ag NPs	5.61	0.2 × 10^10^	-	^40^
Er -doped TiO_2_ NWs	3.52	8.72 × 10^10^	-	^7^
n-type ZnO/p-type NiO	2.02	-	-	^41^
**MgZnO TF/NiO NPs**	**6**	**1.45 × 10** ^**10**^	**~10** ^**2**^	**This work**

Significant values are in bold.

## Conclusion

In this study we have successfully fabricated NiO NPs over MgZnO TF using RF sputtering technique and demonstrated its memory application. The variation in accumulation capacitance was observed from capacitance (C) – voltage (V) characteristic at varying frequencies with larger capacitance in low frequency region. The low interface state density (D_it_) of MgZnO TF/NiO NPs device was found to be 1.45 × 10^10^ eV^− 1^ cm^− 2^ at 1 MHz signifying a low lattice mismatch and better interface quality between MgZnO TF and NiO NPs. A large memory window, good endurance, and retention was obtained, which demonstrate a stable and efficient charge storage capacity of the device. Moreover, a good resistive switching ratio of the device also indicate its potential application in resistive memory devices. The results obtained in this study indicate that the device may be a good fit for nanoscale capacitive and resistive memory application.

## Data Availability

Data will be made available on reasonable request from the corresponding author.
